# Browsing through sealed historical manuscripts by using 3-D computed tomography with low-brilliance X-ray sources

**DOI:** 10.1038/s41598-018-33685-4

**Published:** 2018-10-18

**Authors:** Daniel Stromer, Vincent Christlein, Christine Martindale, Patrick Zippert, Eric Haltenberger, Tino Hausotte, Andreas Maier

**Affiliations:** 1 Pattern Recognition Lab, Computer Science, Friedrich-Alexander-Universität Erlangen-Nürnberg, Erlangen, Germany; 2 Machine Learning and Data Analytics Lab, Computer Science, Friedrich-Alexander-Universität Erlangen-Nürnberg, Erlangen, Germany; 3 Institute of Manufacturing Metrology, Mechanical Engineering, Friedrich-Alexander-Universität Erlangen-Nürnberg, Erlangen, Germany

## Abstract

Severely damaged historical documents are extremely fragile. In many cases, their secrets remain concealed beneath their cover. Recently, non-invasive digitization approaches based on 3-D scanning have demonstrated the ability to recover single pages or letters without the need to open the manuscripts. This can even be achieved using conventional micro-CTs without the need for synchrotron hardware. However, not all manuscripts may be suited for such techniques due to their material and X-ray properties. In order to recommend which manuscripts and which inks are best suited for such a process, we investigate six inks that were commonly used in ancient times: malachite, three types of iron gall, Tyrian purple, and buckthorn. Image contrast is explored over the complete pipeline, from the X-ray CT scan and page extraction to the virtual flattening of the page image. We demonstrate, that all inks containing metallic particles are visible in the output, a decrease of the X-ray energy enhances the readability, and that the visibility highly depends on the X-ray attenuation of the ink’s metallic ingredients and their concentration. Based on these observations, we give recommendations on how to select the appropriate imaging parameters.

## Introduction

Historical documents are relics of the past, containing information about long-forgotten times. Due to aging or external influences, many of these cultural assets cannot be further investigated as they are too fragile to open or page-turn, so their valuable contents remain hidden. In 1750, Karl Weber discovered the ‘Villa dei Papiri’ near Herculaneum, where more than 1800 papyri were found carbonized by the eruption of the Mount Vesuvius in 79 AD^1,2^. Although the volcanic eruption preserved the scrolls, it is impossible to unroll them without causing damage. There are also less extreme examples, such as in the Germanisches Nationalmuseum (Nuremberg, Germany), where pages in the book fold area are stuck together due to aging. In addition, external influences such as floods, fire or war may continue to generate more and more sealed documents. A recent example is the Duchess Anna Amalia Library fire in 2004 (Weimar, Germany) where 62000 volumes were severely damaged, not only by fire, but also by the water used during fire-fighting^3,4^.

Historical books basically consist of three parts: the cover, the pages and the ink(s). Book covers are diverse as they were hand-made and sometimes very luxurious, using materials ranging from leather or wood to ivory, gold or silver^5,6^. Parchment, papyrus or handmade paper was used as writing medium, where the latter was established in the Middle Ages^7^. Since the Roman Empire, iron gall ink has been widely used for writing^8–10^. Thomas Jefferson’s ‘Declaration of Independence’, Goethe’s ‘Faust’, Mozart’s ‘The Magic Flute’ and even some of Rembrandt’s sketches^11^ are just a few examples which were written or illustrated with iron gall ink. Due to its indelible nature, it is even partly still in use today. The Germanisches Nationalmuseum has a collection of historical works consisting of 3380 manuscripts, dating from the early Middle Ages to the early 20 century, where about 95 percent of them were written with iron gall ink. The main iron gall ink ingredients are tannic acid, gum arabic and iron salt (FeSO_4_)^12,13^. Since this ink could only be used to write in black, other inks were invented. Malachite ink is based on metallic particles with its greenish color caused by Cu_2_CO_3_(OH)_2_^14,15^. Tyrian purple ink (also called ‘Royal purple’) used the mucous secretion of sea snails containing Bromine^16^ as dye, whereas buckthorn berries were used to achieve yellow and ocher inks^17^.

In 2007, Bergmann *et al*.^18^ used X-ray fluorescence imaging to reveal hidden writings written with iron ink on pages of the Archimedes palimpsest. The same method was used to differentiate between two inks of the Qur'ān palimpsest^19^, where the erased ink had a different material composition to that of the newer ink. However, this method can only be applied directly to a specific page and requires the book to be opened. Where opening the book is not possible, 3-D X-ray CT imaging can be employed, as in this work. This method has not only been used for human medicine but also for archaeological purposes such as scanning cultural heritages^20–22^. Previous works show that conventional micro-CT systems can deliver good results by exploiting the various material characteristics of historical ink and paper^23–28^. If the ink composition has a higher attenuation than the paper’s cellulose, the ink is recognizable in the volume of an X-ray CT scan^23^ enabling the contents of a book to be known without the need to open a fragile manuscript. This is most effective in cases where metallic particles were present in the ink. Even ink that has been erased and overwritten can still be recognized if some particles penetrated deeper layers of the paper are still present^29,30^. With its high resolution, 3-D X-ray CT is well suited to digitize historical documents. A disadvantage of this method is the X-ray radiation which could accelerate the aging process of cellulose when performing a scan^31,32^. However, the impact of this X-ray radiation on the dry paper and ink is still very limited^33^. The dose can also be kept to a minimum while preserving the relevant information, as shown in our previous work^34^.

One method which does not expose the document to ionizing radiation is 3-D Terahertz imaging^35,36^. A major drawback of this technique is its limited penetration depth, allowing only a few pages to be digitized. Until now, Terahertz imaging has not been evaluated on real historical documents where the effect of the metallic particles in the ink reflecting the Terahertz waves is unknown. A second technique is X-ray phase contrast imaging capable of scanning manuscripts, books or archaeological relics^37–39^. However, this technique is neither widely accessible nor mobile. Libraries would have to transport their valuable books and documents to the measurement centers which is costly due to security and insurance reasons. Whereas micro-CT systems could be mounted in libraries because they are more mobile than phase contrast hardware.

This paper describes the complete pipeline for an X-ray CT digitization of a book which cannot be opened anymore. Six different inks (metallic and non-metallic) were used to generate writings and evaluate their visibility. First, we performed three 3-D X-ray micro-CT scans with different parameter sets and used a fast and accurate 3-D reconstruction technique leading to 3-D volumes of the book. Next, we applied an algorithm to these volumes to extract and map all individual pages into 2-D^40^ for further investigation. Finally, we compared the three different scans with regard to the visibility of the writings. To the best of our knowledge, the entire digitization pipeline for such books has not been analyzed in detail. The process contains many variable options and parameters: the book’s components, 3-D scan parameters, 3-D reconstruction approaches, page extraction and 2-D mapping. In this work, we provide details on each step and expose the limitations of each proposed technique. We also highlight the new challenges that will arise due to the resulting 2-D pages of the X-ray volumes, as this output fundamentally differs from the state of the art high resolution camera images currently used within the field of document digitization.

## Material

**Figure 1:**
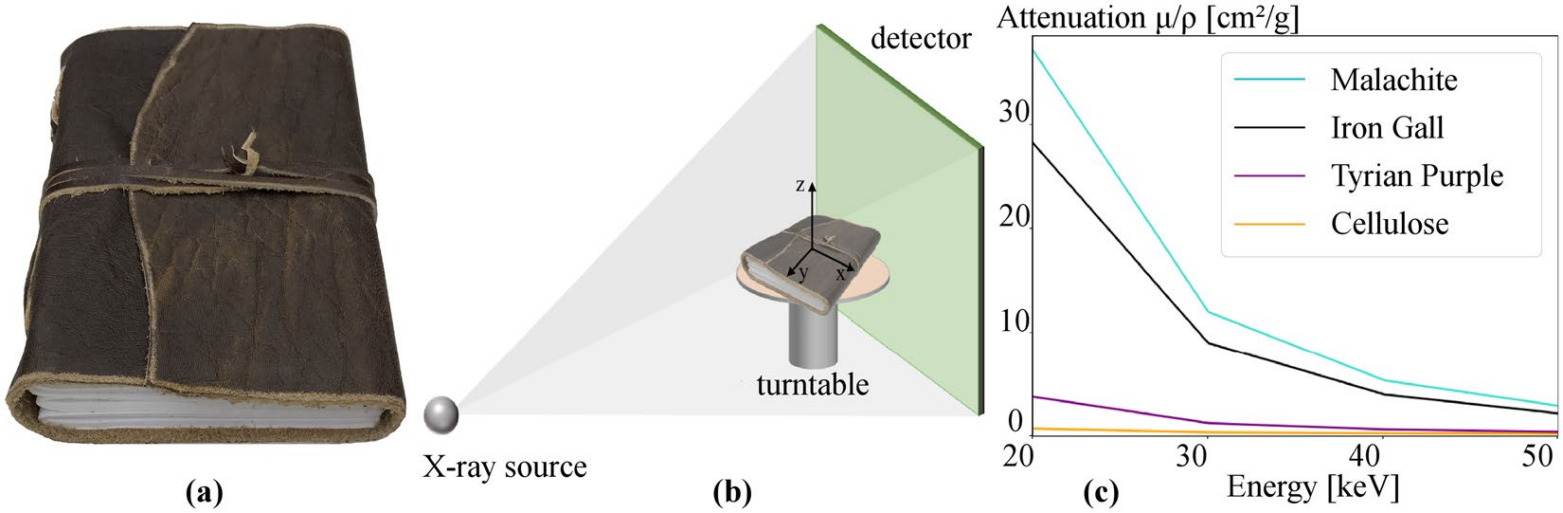
(**a**) The book consisting of 56 handmade paper pages covered in buffalo leather. (**b**) Schematic of the book placement within the scanner with the cover orthogonal to the rotation axis *z*. While the turntable rotates, a set of projection images are acquired, from which a 3-D volume is calculated. (**c**) X-ray attenuation plot for the different inks and paper–malachite ink has the highest attenuation, followed by iron gall and Tyrian purple. As buckthorn ink and paper are both made of cellulose their absorption is the same.

Before working with real historical documents, every process within the digitization pipeline must be optimized. This ensures that only one scan is performed with optimal parameters thus minimizing radiation exposure to the document. Therefore, we made use of a self-made book with the following dimensions: 17 × 13 × 3 cm (L × W × H), as shown in Fig. 1(a). It consists of a buffalo leather cover and 56 pages of handmade paper with a page thickness of about 150 μm. The focus of this study is to investigate the visibility of six different inks in the X-ray CT volume: three different iron gall inks, malachite ink, Tyrian purple ink and buckthorn ink. It should be mentioned that we used two different iron gall inks and additionally produced a third version by adding more FeSO_4_ to one of the original inks. This enables the assessment of the effect of the number of iron particles on the visibility of the output. Malachite and Tyrian purple ink were chosen because they consist of different metallic components, while buckthorn ink is only cellulose-based.

**Figure 2:**
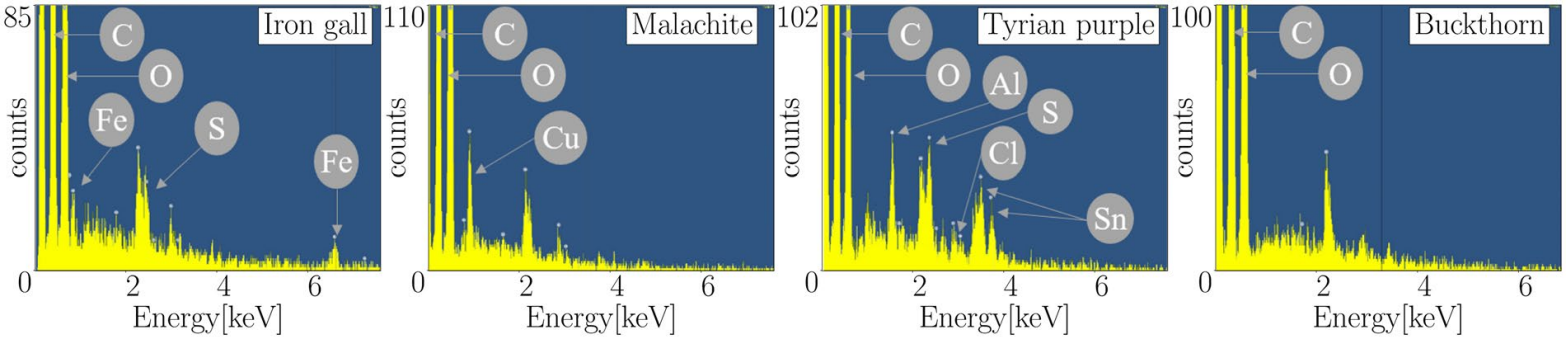
Energy-dispersive X-ray spectroscopy of the inks: malachite ink consists of Cu. For all three iron gall inks, the elements Fe and S were detected (one exemplary ink is shown). Tyrian purple ink consists of Al, S, Cl and Sn. Buckthorn ink does not consist of metallic particles, but only C and O. All spectra show C and O which is caused by the cellulose of the paper.

Initially, we performed an energy-dispersive X-ray spectroscopy (EDS) in the range of [0, 8] keV for each ink to analyze the present materials, as shown in Fig. 2. EDS is based on X-ray fluorescence and is capable of obtaining the elemental composition of materials, from which the ink’s X-ray characteristics can be derived. Diverse elements have varying X-ray attenuations for different X-ray energies allowing the optimal scanning energy to be determined. Furthermore, performing an EDS of the ink in advance may give insight into whether a CT scan of the given document is suitable. In a real setup, where the document cannot be opened anymore, such a measurement cannot be acquired. However, we wanted to ensure that the used materials were not contaminated by other materials affecting the attenuation. The EDS revealed that malachite ink consists of Copper (Cu), Carbon (C) and Oxygen (O) and all iron gall inks consist of Iron (Fe), Oxygen (O) and Sulfur (S). The analysis of Tyrian purple ink showed that Carbon (C), Oxygen (O), Aluminum (Al), Sulfur (S), Chloride (Cl) and Tin (Sn) were present. It should be mentioned that while this ink was formerly made from the secretions of sea snails, producers currently substitute the ingredients with cheaper ingredients. In comparison to the previous mentioned inks that consist of metallic particles, we also evaluated buckthorn ink where the EDS detected only Carbon (C) and Oxygen (O).

As X-rays are used to digitize the document, the X-ray attenuation coefficients *μ*′ = *μ*/*ρ* were calculated for all used inks based on the NIST XCOM database^41^, as illustrated in Fig. 1(c). The energy range was chosen based on previous work^24^ in which we showed that the higher the attenuation difference of the paper and ink, the better the visibility of writings in the output volume. One can observe, that malachite has the highest attenuation, followed by iron gall ink and Tyrian purple. Buckthorn ink, as well as paper, only consist of cellulose, resulting in the same X-ray attenuation coefficient. Further increasing the X-ray energy only leads to slight differences of the materials’ X-ray attenuation coefficients thus the writings would vanish in the output.

## Methods

### The volumetric scan

The scans were performed on a 3-D X-ray micro-CT using cone-beam geometry. The calculations were made by using the CONRAD framework^42^. Figure 1(b) shows the schematic structure of the scan. The book is placed on a turntable, where a projection image is acquired at each angular step. From the resulting set of projections, a 3-D volume is calculated by applying an appropriate reconstruction algorithm. The book’s front cover lies orthogonal to the rotation axis *z*, aiming to achieve a balanced penetration length of the X-ray beams through the object^23^ for artifact reduction. We performed three 360° scans with 1800 projections. As mentioned above, the X-ray energy should be minimized and therefore we chose 30 kV, 40 kV and 50 kV, where 30 kV constitutes the scanner’s lowest configurable energy. For the 50 kV scan, we used an additional copper pre-filtration of 0.25 mm to narrow the polychromatic X-ray spectrum^43^. The drawback of such low X-ray energies is increased noise, so a trade off between noise and ink visibility is necessary. By using 30–50 kV, a tube current of 3 mA and an exposure time of 2 s, the signal-to-noise ratio (SNR) is still acceptable. The ratio of source-to-object (SOD) (710 mm) to source-to-detector (SID) (1377 mm) distances and the detector pixel size of 0.2 × 0.2 mm^2^ results in a voxel size of 103 × 103 × 103 μm^3^.

### 3-D X-ray CT reconstruction

The state-of-the-art 3-D reconstruction approach in cone-beam CT is the Feldkamp, Davis and Kress algorithm^44^ where the projection images are cosine weighted, ramp filtered and back projected to receive the final 3-D volume. According to Tuy’s condition^45^, the FDK algorithm delivers exact results only in the central plane. The greater the cone-angle *γ* and the further the slices are from the centre, the more the result suffers from cone-beam artifacts due to the insufficient circular orbit sampling^46–48^. We tried to minimize these artifacts with a horizontal book placement, reducing the effective worst-case cone-angle ${\gamma }_{max}=\arctan \,(0.5\cdot {H}_{book}\cdot {d}_{min}^{-1})$ to a minimum. *H*_*book*_ denotes the book’s height and *d*_*min*_ denotes the minimum distance between the X-ray source and book, calculated by *d*_*min*_ = *SOD* − 0.5 · *l*_*book*_. Here, *l*_*book*_ denotes the book’s diagonal length (214 mm). This yields *γ*_*max*_ ≈ 1.43° and *γ*_*min*_ = arctan (0.5 · *H*_*book*_ · *SOD*^−1^) ≈ 1.21° at the rotation center. Such small cone angles result in more than 99 percent of the Radon sphere being sampled^49^ enabling in a very high reconstruction accuracy. In addition, the ink is more dispersed along the plane of the pages and conversely, the ink lies in regions where adjacent pages are very close to each other in the perpendicular page plane. This fact makes a horizontal document placement in the scanner more favorable due to artifact reduction.

### Page extraction and 2-D mapping

**Figure 3:**
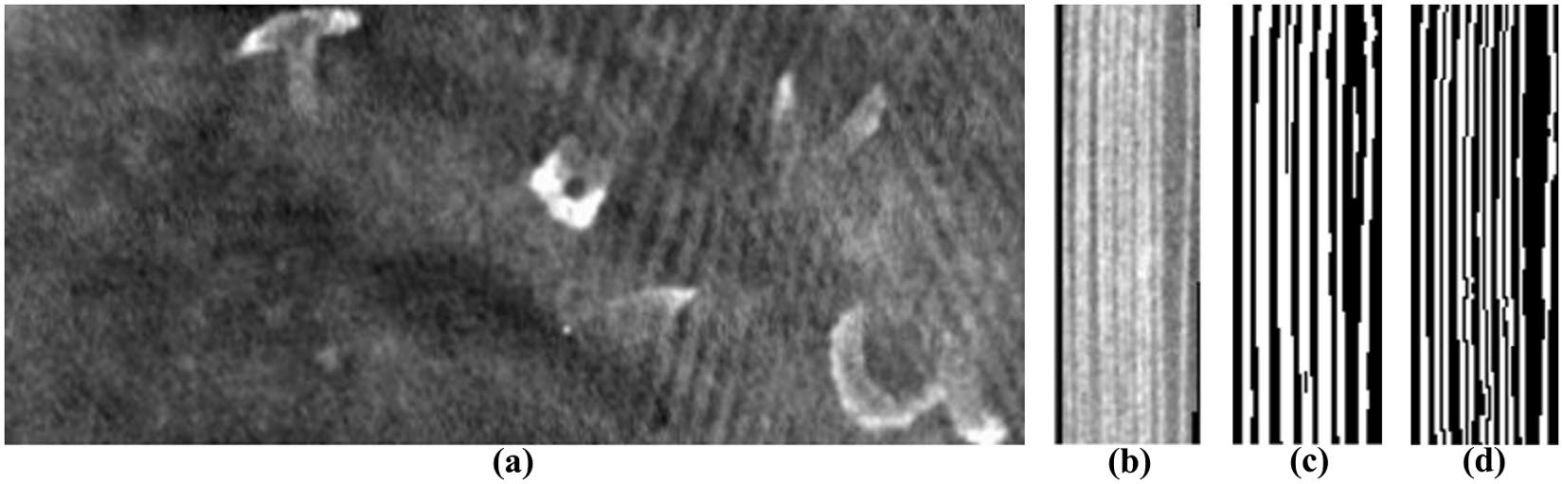
(**a**) Exemplary X-ray volume *xy*-slice of the scanned book: The high resolution and the orientation of the pages in the volume make it impossible to read each page separately with the naked eye. Multiple pages are present in one slice such that a virtual flattening is necessary. (**b**) *yz*-slice of the volume–the pages are separated by air gaps and look like small vessels. (**c**) The same *yz*-slice after applying the volume binarization step–the pages are binarized but still contains overlaps and gaps. (**d**) The final output after page segmentation for the *yz*-slice of (**b**)–overlapping pages are separated and gaps are closed.

**Figure 4:**
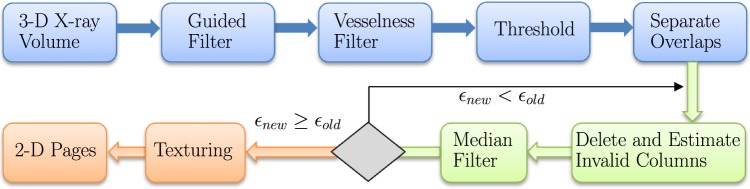
Volume processing pipeline for automatic page extraction. Within pre-processing (blue boxes), the volume is denoised, binarized and overlapping pages are separated. Next, the pages are smoothed and holes are filled iteratively (green boxes). A texturing step stores the maximum value along the height of a page in a 2-D image resulting in the final 2-D mapped page (orange boxes).

The greatest challenge after performing the 3-D X-ray micro-CT scan is to handle the 3-D volume. Due to the high resolution and the thin and wavy pages, which are also squeezed together, the separation of pages within the 3-D volume is not trivial. Each page has a thickness of around 150–200 μm resulting in around 1–3 voxels with the given voxel size. The rather low X-ray energies simultaneously increased the noise level. Figure 3(a) shows an exemplary *xy*-slice of a volume where multiple pages are present such that the investigation of a single page is not possible without virtually flattening the pages. As a manual segmentation is too time-consuming, a fully-automatic page-extraction method was developed^40^ to extract and map the pages to 2-D. The pipeline of the complete algorithm is illustrated in Fig. 4 and can be broken down into three steps:

- *Volume binarization (blue boxes)*: Initially, the volume **V** is filtered using a Guided-filter^50^ for edge-preserved smoothing. Due to the pages appearing like small vessels within the volume, illustrated in Fig. 3(b), we utilized a vessel segmentation technique for volume binarization. Vesselness filtering proposed by Frangi *et al*.^51^ is applied on every *yz*-slice resulting in **V**_vessel_. The Vesselness algorithm first filters an image ***I*** with multiple scales *s*_*i*_, using a Gaussian filter. Then, the eigenvalues *λ*_1_, *λ*_2_ (|*λ*_1_| ≥ |*λ*_2_|) of the filtered image’s Hessian matrices are calculated for each pixel. The eigenvalues indicate the principal directions of an image’s second order structure, leading to the smallest curvature along the page. The blobness measure $R={\lambda }_{2}\cdot {\lambda }_{1}^{-1}$ is calculated and is close to 0 in case of a page being present. Next, $S=\sqrt{{\lambda }_{1}^{2}+{\lambda }_{2}^{2}}$ is computed. When a pixel is part of a page, *S* will become larger since at least one of the eigenvalues will be large. A page is enhanced by evaluating
  1 $${{\boldsymbol{V}}}_{{\rm{v}}{\rm{e}}{\rm{s}}{\rm{s}}{\rm{e}}{\rm{l}}}=\{\begin{array}{cc}0 & ,\,{\rm{i}}{\rm{f}}\,{\lambda }_{1} > 0\\ \exp (-\frac{{R}^{2}}{2{\beta }^{2}})(1-\exp (-\frac{{S}^{2}}{2{\gamma }^{2}})) & ,\,{\rm{o}}{\rm{t}}{\rm{h}}{\rm{e}}{\rm{r}}{\rm{w}}{\rm{i}}{\rm{s}}{\rm{e}}\end{array},$$
  where *β* and *γ* are control parameters depending on the grayscale intensity of the image. The result of Eq. (1) yields a probability map for the likelihood of a certain voxel being part of a page (**V**_vessel_ → 1) or air (**V**_vessel_ → 0) and subsequent global thresholding (**V**_vessel_ ≥ 0.1 = 1, **V**_vessel_ < 0.1 = 0) binarizes the volume. The result of this step is shown in Fig. 3(c). Subsequently, the total number of pages *N* and the mean page thickness $\bar{p}$ are estimated by counting all pages and calculating their thicknesses for all rows in every *yz*-slice. Given $\bar{p}$, overlapping pages are separated by splitting pages in the center that are thicker than $1.5\cdot \bar{p}$. Fig. 3(d) shows the result of the entire binarization and separation process.
- *Page segmentation (green boxes)*: Within this process, two steps are repeated until a stop criterion is reached. First the actual number of detected pages *N*_*y*_ are counted for all rows *Y* of a *yz*-slice; if *N*_*y*_ differs from *N*, the corrupted row number *y* is stored in a list. After performing this step for all *yz*-slices, the corrupted rows are replaced by the nearest uncorrupted row with the same *y* indices. The total number of corrupted rows is stored as *ε*. The *xy*-slices of the volume are median-filtered to remove outliers and to close gaps. These steps are repeated until the newly calculated *ε* is equal or greater than the old, i.e. that no further enhancement is possible because the number of smoothed rows does not change.
- *Texturing and 2-D mapping (orange boxes)*: The final step is to sample the page at each point (*x*, *y*) along *z*-direction and store the maximum value in a 2-D image. The maximum is considered because the writings appear brighter in the image than air or paper. As the pages can be wavy, their curvature has to be considered. Therefore, sampling along the line perpendicular to the page’s central line considers the waviness of the pages and yields more exact results than straight sampling.

### Contrast ratio estimation

Before measuring objects with an X-ray CT scan, the expected contrast C of the given materials compared to vacuum can be estimated from the scan parameters and the mass absorption coefficients ${\mu ^{\prime} }_{m}$ of each material *m* (c.f., Fig. 1(c))^52^. C is given by

2 $${\rm{C}}=1-\exp \,(\,-\,{p}_{m})=1-\exp (\,-\,{\mu ^{\prime} }_{m}{\rho }_{m}{d}_{m}),$$

where *p*_*m*_ denotes the attenuation of material *m*, and *d*_*m*_ the material’s thickness. In a probabilistic sense, this is equivalent to the percentage of photons that will be absorbed within the material. In the multi-material case, the individual material attenuations are summed up over all materials *M* according to

3 $${{\rm{C}}}_{{\rm{mm}}}=1-\exp \,(\,-\,{p}_{mm})=1-\exp (-\,\sum _{i}^{M}\,{p}_{i}).$$

To obtain the final contrast ratio, we divide the multi-material C_mm_ of ink and paper by the paper-only case:

4 $${{\rm{CR}}}_{{\rm{est}}}=\frac{{{\rm{C}}}_{{\rm{mm}}}}{{{\rm{C}}}_{{\rm{paper}}}}\mathrm{.}$$

The higher CR_est_, the better the contrast of the ink. This is only an approximate estimate neglecting the effects of noise. Under almost noise-free conditions it is an indicator, whether an X-ray scan will lead to reasonable results.

## Results

Each scan took about 2 h where the 3-D reconstruction was performed on-line resulting in a scan and reconstruction time of about 2 h 10 min. The runtime of the extraction algorithm was approximately 15 min for each volume. For the three scans, the resulting 2-D mapped pages were compared with regard to the visibility of the inks. Before extracting the pages, the book cover was deleted manually within every volume to enhance the algorithm’s output.

### Book placement

**Figure 5:**
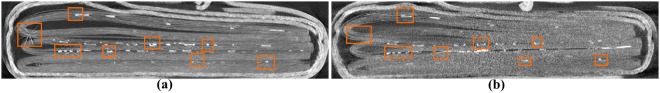
3-D X-ray CT reconstructed volume *xz*-slice with different book placement where the orange boxes denote the areas of interest. (**a**) Vertical book placement–the penetration beam length varies due to the placement resulting in metal artifacts that penetrate the pages. (**b**) Horizontal book placement with cover orthogonal to the rotation axis–no metal artifacts are visible.

First, the influence of the book placement is evaluated. We performed a scan with a horizontal book placement setting the cover orthogonal to the rotation axis and compared it to a scan with a vertical placement. Figure 5 shows the results for an exemplary central *xz*-slice. Figure 5(a) depicts the *xz*-slice of the scan with a vertical book placement. The varying penetration beam length caused metal artifacts (oranges boxes) where the ink is located. As those artifacts only appear in beam direction, the separability of the pages is negatively affected. With the horizontal book placement scan, the artifacts do not appear, cf. Fig. 5(b). The increased noise is due to the fact that the vertical scan was a large volume scan^53^ with a voxel size of around 59 × 59 × 59 μm^3^.

### Qualitative analysis

**Figure 6:**
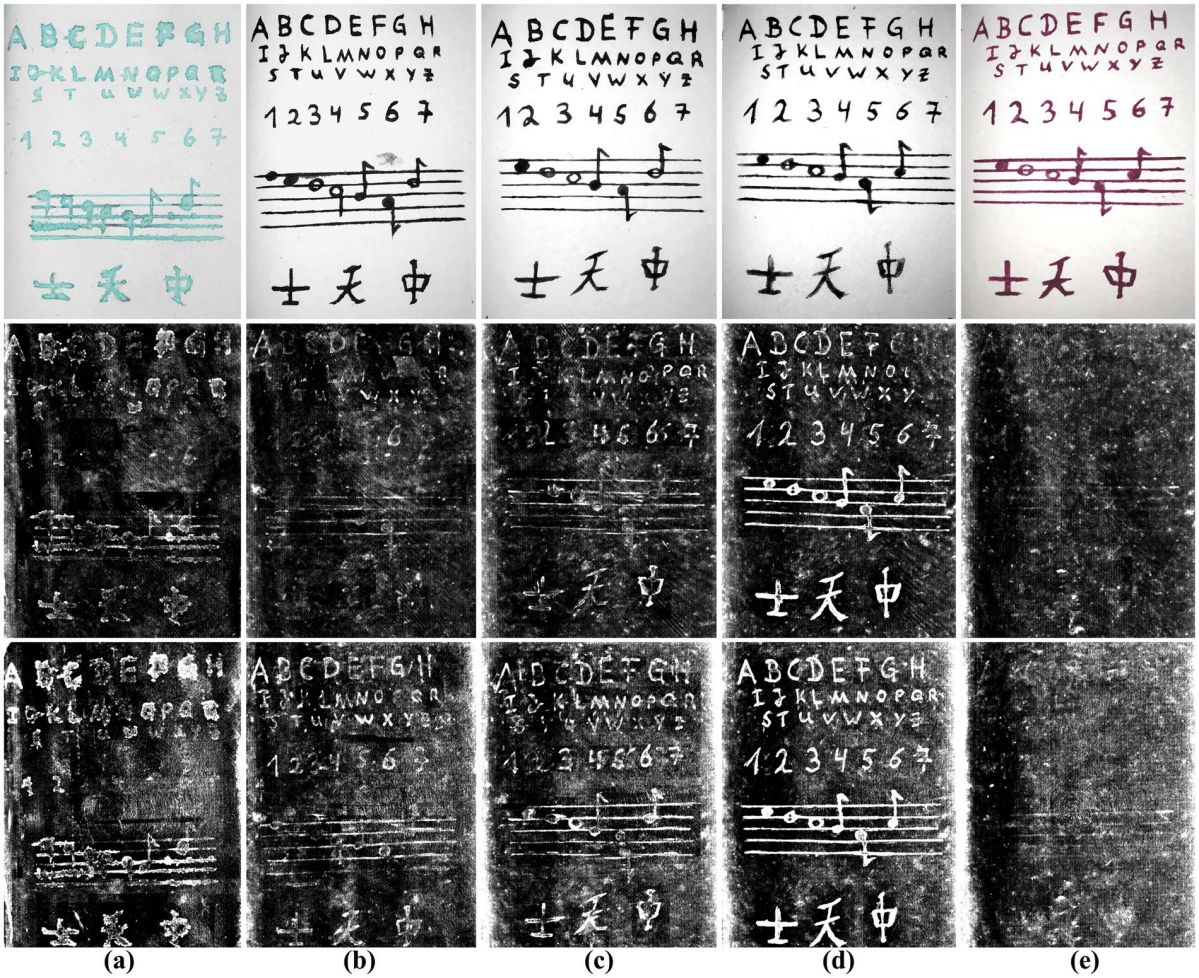
Photographs of the original pages are shown in the top row–(**a**) malachite ink, (**b–d**) iron gall ink 1–3. (**e**) Tyrian purple ink. The center row shows the reconstructed and 2-D mapped pages of the closed book for the 50 kV scan, the bottom row the 30 kV scan output. Tyrian purple ink has a small attenuation and the writings can be seen only slightly compared to iron gall and malachite ink.

The top row of Fig. 6 shows photographs of the original pages written with (**a**) malachite ink, (**b**–**d**) the three iron gall inks, and (**e**) Tyrian purple ink. The center row of Fig. 6 shows the reconstructed and 2-D mapped pages of the closed book for the 50 keV scan with the copper pre-filter. The bottom row shows the 30 scan output after page extraction.

Most of the malachite ink writings can be identified within all performed scans. The result for the malachite ink shows some artifacts in the center of the output. This is due to the fact that the malachite ink page was placed next to the cover which touched the page in the central region, resulting in the obvious dark areas. However, this does not affect the visibility of the writings themselves. This is also true for the iron gall ink as well, where the writings, numbers and symbols are visible on the extracted pages, except for the central regions where they are less visible. When comparing the three iron gall inks, we observe that iron gall ink 3–which is the ink with additional FeSo_4_–has brighter intensities than the original inks. This confirms our assumption that an increased amount of metallic particles increases the writings’ visibility. The Tyrian purple ink writings are only slightly visible, which is due to the lower X-ray attenuation coefficient. Here, only a few symbols can be identified. Buckthorn ink could not be differentiated from the paper because the attenuation is the same according to Fig. 1(c). Hence, we omitted the output results.

For calculating CR_est_, the paper thickness *d* was set to 200 μm referring to the page thickness while the ink thickness was set to 2 μm. The estimated results for all inks show, that the lower the selected tube energy, the better the contrast between ink and paper. Next, we measured the intensity difference of the inks and paper. Therefore, we manually segmented the Chinese symbols on the bottom of the page, calculated the mean intensity of this area *m*_1_ and subtracted the mean of the surrounding paper-only area *m*_2_. From these values we determined the measured contrast-to-noise ratio CR_msr_ = |*m*_1_ − *m*_2_| · ${\sigma }_{0}^{-1}$, where *σ*_0_ denotes the standard deviation of the pure image noise. The results for all scans are shown in Table 1. The 50 kV scan has the lowest values, followed by the 40 kV scan. The 30 kV scan shows the best results for all inks. Furthermore, we can observe that iron gall ink 3 (IG3) has the best contrast measure caused by the additional iron-II-sulfate. The copper based malachite ink has a higher intensity difference than the original iron gall inks (IG1, IG2) and Tyrian purple has the lowest visibility of all inks. The measured CR_msr_’s are generally consistent with the estimated CR_est_’s for all inks.

**Table 1:** Ink contrast evaluation for the three performed 3-D X-ray CT scans.

Tube energy	Malachite		Iron Gall				Tyrian Purple	
	CR_est_	CR_msr_	CR_est_	IG1: CR_msr_	IG2: CR_msr_	IG3: CR_msr_	CR_est_	CR_msr_
50 kV	1.74	0.90	1.49	0.32	1.24	4.23	1.03	0.07
40 kV	2.19	1.55	1.78	0.92	1.25	4.33	1.04	0.22
30 kV	2.98	**1**.**76**	2.32	**1**.**12**	**1**.**55**	**4**.**68**	1.06	**0**.**45**

All writings, symbols and numbers were marked. From the mean value of the marked area and the page-only mean value, the contrast ratio (CR_msr_) was determined for every scan and ink (higher values indicate improved visibility). The best contrast for all inks was measured with the 30 kV scan. The highest attenuation was measured for the iron gall ink with additional FeSO_4_ (IG3), followed by malachite ink, the original iron gall inks (IG1, IG2) and Tyrian purple. The estimated contrast ratio CR_est_ and CR_msr_ are generally consistent.

### Chromatographic effect of ink

**Figure 7:**
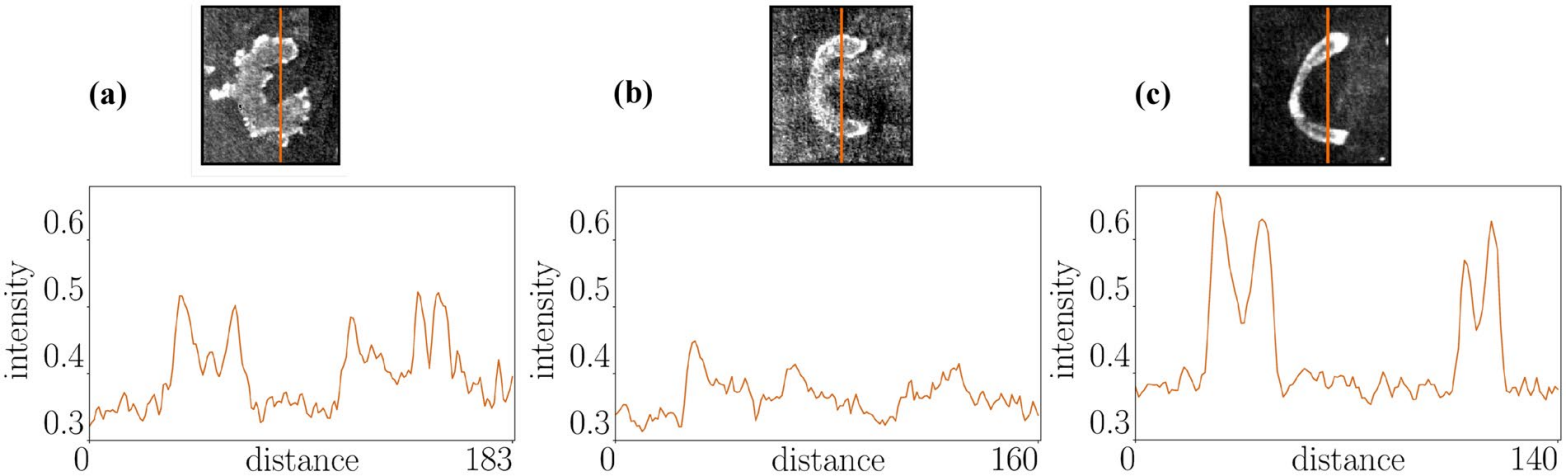
Zoom on letter ‘C’ from the 30 kV scan for three inks inks. (**a**) Malachite ink, (**b**) iron gall ink 1, (**c**) iron gall ink 3. A plot along the orange line shows, that the intensities at the border regions are higher than in the central region. This can be explained by the chromatographic effect when the spread of the metallic particles is stopped within the paper in border regions resulting in a higher concentration of these particles and thus higher X-ray attenuation.

The top row of Fig. 7 shows a snippet of the letters ‘C’ for malachite ink (Fig. 7(a)), iron gall ink 1 (Fig. 7(b)) and iron gall ink 3 (Fig. 7(c)) scanned with 30 kV. We can observe that the X-ray attenuation increases towards the edges of the letter. This can be explained by the chromatographic effect. Unlike papyrus or parchment, handmade paper absorbs most of the ink. When the ink spreads out in the paper, the transport of the liquid portion is faster while the metallic particles slow down until they completely stop and accumulate at a certain region. This results in a higher X-ray attenuation in the border regions. This is emphasized by the plots along the orange lines shown in the bottom row of Fig. 7. The intensities around the border regions are two to three times higher than the background, whereas in the central region the intensities are about 1.5 times higher. Additionally, the appearance of the letters differs from ink to ink. While the iron gall ink writings look smooth at the borders, the malachite ink has a more grainy structure.

## Discussion

The scans of the self-made book, consisting of 56 pages of handmade paper, a buffalo leather cover and six different inks, showed very promising results. We showed that the book placement plays an important role for the quality of the output. We proposed an horizontal book placement such that the cover is orthogonal to the system’s rotation axis. This improved the image quality compared to a vertical setup showing severe artifacts and is furthermore easier to mount for fragile documents.

In order to expand these results, the effect of different sizes of books needs to be addressed. For larger books, the scan parameters need to be adapted because the X-ray attenuation might be too high to detect a signal. Also the voxel size will increase with regard to the scanners flat panel detector, however, this can be compensated by configuring a smaller source-to-object distance. Initially, we tested the algorithms on a small book having a dimension of 4 × 4 × 0.5 cm^3^ ^40^. We were able to reduce the voxel size to around 30 × 30 × 30 μm^3^, whereas the pages had a thickness of approximately 150 μm resulting in 4–6 voxels/pages. With the larger book presented in this study, the voxel size was increased to 103 × 103 × 103 μm^3^ such that a page is covered by only 13. This is the minimal requirement to separate pages in the output volume. By using a large volume scan or with future improved scanner resolutions, the output quality will improve too.

We showed that reducing the X-ray energy of the scan improved the visibility of the writings but simultaneously increased noise. This noise could be reduced by averaging over multiple projections, with the drawback of an increased radiation dose. To counter this, other reconstruction approaches, such as a total-variation-regularized reconstruction, can be used. We showed in an earlier work^34^, that for the aforementioned small book, the number of projections can be reduced to a minimum using a short scan trajectory instead of a full-circle scan, combined with iterative reconstruction techniques.

Until now, we created a database consisting of thirty 3-D X-ray book scans. We used three different scanners, six different books and varying scan parameters. The data was processed by the page extraction algorithm and will provide a database for training-based algorithms, to increase the accuracy of the segmentation, e. g. an automated separation of the cover and page or for handling even noisier data due to different document dimensions or scan parameters. So far we have not tested any double-sided pages.

We showed that ink has higher attenuations at the script edges due to the chromatographic effect of the ink in handmade paper. Also in central areas of the writings, higher grayscale intensities compared to the papers’ where obtained. The ink visibility highly depends on the ink’s metallic ingredients and the quantity of metal particles. The higher the concentration of the metal and the higher the X-ray attenuation of the element, the better the visibility. The CRs of the combined materials are in general consistent with the measured CRs. We were able to separate the original iron gall inks from the malachite ink when comparing the mean intensity of a certain letter which could help to distinguish between different inks. For example, the first letter of a new chapter was often ornamented and highlighted with a different color than the rest of the chapter. With the varying intensity in the X-ray volume, we can separate the inks and highlight other inks, too.

Based on our experiments, we recommend to use a tube current of ≥3 mA with an exposure time ≥2 s (6 mAs). Lower mAs levels reduced the CR and thus the visibility of the ink. The higher the concentration of metallic particles in the ink, the higher the X-ray energy which can be selected simultaneously suppressing the noise level. An energy range of [20, 40] kV showed up to be a good trade off between signal and noise in the output.

In comparison to a synchrotron setup, there are several disadvantages of standard 3-D X-ray CT systems. Due to brighter and more monochromatic X-rays, the synchrotron setup achieves an improved SNR, sensitivity and spatial resolution. This can be useful for an improved page separation and for small and degraded writings appearing in realistic documents. While standard CT systems often have energy limitations, the X-ray energy can be adjusted more easily, allowing the optimization of the scan parameters. As the proposed estimation of the contrast is based on ideal conditions (e. g. monochromatic X-ray source, noise-free), the synchrotron setup’s contrast should shift towards the calculated values and hence improve the results. Conversely, conventional X-ray CT systems are more mobile than synchrotron systems as they can be brought to libraries delivering sufficient results for a digitization.

We are aware that the book is not a real historical document that suffers from aging. Furthermore, there is a wide range of materials that were used to built books such as wooden covers or parchment, which might behave differently from our materials. The proposed digitization pipeline is intended to provide a basis for future research in this research area. A practical example where this digitization process could be implemented is the Germanisches Nationalmuseum in Nuremberg, Germany. One ongoing case is a book where pages are stuck together at the area of the book fold. In this case, the restorer has to trade off the possible damage to the writings within the manual process versus the damage from the CT. As the manuscript is written with iron gall ink, the conservator could consider digitizing the manuscripts by applying an initial 3-D X-ray micro-CT scan, allowing any parts of book’s writings which may be destroyed by the manual conservation process to be restored by using the 3-D X-ray CT volume.

## Summary

In this work we presented a non-invasive approach capable of recovering information from manuscripts or books that cannot be opened or page-turned anymore.

Instead of using immobile synchrotron or phase contrast hardware, an X-ray micro-CT system was used to scan the document. We showed that book placement plays an important role for improving the output quality, such as reducing metal or cone-beam artifacts. Furthermore, we make a recommendation for a set of scan parameters, based on our experiments, to enhance the ink visibility. As the 3-D volume with its high resolution and wavy, overlapping pages cannot be investigated precisely with the naked eye, a fully automatic page extraction and 2-D mapping algorithm is presented allowing one to browse through the book’s pages virtually.

To the best of our knowledge, the complete digitization pipeline from scanning to 2-D mapping of the pages was analyzed for the first time in this work. The process consists of many variable parameters requiring careful consideration. To create a realistic simulation, we employed handmade paper and investigated six commonly used historical inks made of different materials with varying X-ray properties. An EDS measurement revealed that the most inks consisted of the desired metallic elements. The measurement of the inks visibility were generally consistent with the estimations and could be further improved with a synchrotron setup.

This study shows many possibilities for the research in the field of digitization of historical documents. allowing the reading of permanently closed books and the revealing of long-forgotten information from past times. Further studies must be conducted to investigate more inks which are visible within X-ray scans and compare this modality to a synchrotron setup, use the segmented data as a basis for a machine learning page extraction and 2-D mapping approaches and refining the scan parameters for books of greater dimensions. Additionally, our group works on reducing the applied radiation dose to a minimum by simultaneously preserving the written information. The data, the source code and detailed calculations are publicly available at the following: https://www5.cs.fau.de/~stromer/.
